# Genome-wide identification, evolutionary dynamics, and abiotic stress response networks of the magnesium transporter gene family in foxtail millet (*Setaria italica* L.)

**DOI:** 10.3389/fpls.2026.1818797

**Published:** 2026-07-01

**Authors:** Jinchen Yu, Haojie Guo, Jinyue Wang, Cheng Wang, Hongfei Ding, Junying Liu, Chaomin Meng

**Affiliations:** College of Agriculture, Henan University of Science and Technology, Luoyang, China

**Keywords:** abiotic stress response, evolutionary analysis, foxtail millet, genome-wide identification, haplotype analysis, magnesium transporter (*MGT*)

## Abstract

Magnesium (Mg²^+^) is an essential mineral nutrient for plant growth, playing a pivotal role in chlorophyll biosynthesis, enzyme activation, photosynthesis, and ion homeostasis. The magnesium transporters (*MGTs*) from the CorA/MRS2-ALR superfamily are crucial for Mg²^+^ uptake, translocation, and subcellular compartmentalization. Foxtail millet (*Setaria italica* L.), recognized as a drought-tolerant C4 cereal with a streamlined genome and robust environmental adaptability, presents an optimal model for investigating stress resilience and C4 photosynthesis. To date, the *MGT* gene family in foxtail millet has not been characterized. In this study, we identified nine *SiMGT* genes (*SiMGT1*–*SiMGT9*) through comprehensive genome-wide screening. These genes are distributed unevenly across chromosomes 4, 5, 7, and 9. Each *SiMGT* possesses conserved CorA domains and displays unique physicochemical attributes and subcellular localization patterns. Phylogenetic assessments categorized the *SiMGTs* into five distinct groups, indicating a close evolutionary relationship with graminaceous crops such as rice, maize, and sorghum, and suggesting functional conservation. Expression profiling highlighted that *SiMGT1*, *SiMGT3*, *SiMGT6*, *SiMGT7*, and *SiMGT8* are consistently highly expressed. Notably, *SiMGT7* is predominantly expressed in leaves, suggesting a potential association with leaf-related or photosynthesis-associated processes, while *SiMGT6* shows preferential expression in panicles, indicating a possible role in reproductive development. When subjected to drought, low temperature, salinity, and ABA treatments, the *SiMGTs* exhibited varied temporal response patterns: *SiMGT7* and *SiMGT9* showed relatively strong responses to drought and cold stress; *SiMGT2* responded prominently to salt treatment; and *SiMGT1*, *SiMGT2*, and *SiMGT3* were rapidly induced at the early stage of ABA treatment. Haplotype analysis pinpointed superior haplotypes (Hap1) of both *SiMGT6* and *SiMGT7*, which showed significant associations with panicle yield traits and stress-related traits, respectively. These findings provide insights into the evolutionary characteristics and expression divergence of the *SiMGT* family and offer candidate genes and molecular markers for future functional studies and the genetic improvement of foxtail millet.

## 1 Introduction

Magnesium (Mg^2+^) is an essential macronutrient for plant growth and development, serving as a structural component of chlorophyll and a cofactor for more than 300 enzymes involved in carbohydrate metabolism, nucleic acid synthesis, and signal transduction (Black et al., 1994; Maguire and Cowan, 2002; Sponder et al., 2013). Disruption of Mg^2+^ homeostasis results in reduced photosynthetic efficiency, stunted growth, yield loss, and increased susceptibility to abiotic stresses (Maguire, 2006). The *MGT* gene family encodes Mg^2+^ transporters that mediate the transmembrane transport of Mg^2+^, including uptake from the rhizosphere, long-distance translocation between tissues, and subcellular distribution among organelles, thereby contributing to plant growth, ion homeostasis, and stress adaptation (Sponder et al., 2013; Chen et al., 2012; Waters, 2011; Bose et al., 2011; Chen et al., 2017; Xu et al., 2015). Recent studies further suggest that Mg-mediated stress adaptation is closely associated with cationic balance and signaling crosstalk, particularly involving Ca^2+^ and K^+^ under adverse conditions (Sarraf et al., 2026; Dölger et al., 2025).

Extensive studies in model plants have elucidated the functional diversity of *MGT* genes. *Arabidopsis thaliana* possesses 10 *AtMGT* members: *AtMGT1*/*7* confer low-Mg^2+^ tolerance, *AtMGT5* is essential for pollen development, and *AtMGT6* maintains chloroplast Mg^2+^ homeostasis (Li et al., 2001; Gebert et al., 2009; Conn et al., 2011; Alexandersson et al., 2004; Sun et al., 2017). Rice (*Oryza sativa*) contains 9 *OsMRS2* genes, among which *OsMRS2-5/6* are highly expressed in root tips and contribute to adaptation to Mg^2+^ deficiency (Saito et al., 2013). More recently, genome-wide analyses of the *MGT* family have been extended to several crop species, including peach, cassava, tobacco, soybean, and wheat, further supporting the structural conservation and functional diversification of MGT/MRS2 transporters across plant lineages (Wu et al., 2022; Zhou et al., 2024; Tan et al., 2024; Akter et al., 2025; Hao et al., 2026).These findings indicate that *MGT* families across different species retain conserved CorA domains, including the GMN motif, and stress-responsive cis-elements such as ABRE and MBS, reflecting a coordinated evolution involving both functional conservation and environmental adaptation.

Despite these advances, three critical knowledge gaps remain: (1) The evolutionary mechanisms driving functional divergence of *MGT* genes between monocots and dicots are not well understood, particularly in non-model crops; (2) Coordinated regulatory networks between MGTs and other ion transporters (e.g., Ca²^+^, K^+^ transporters) during stress adaptation have been poorly characterized in drought-tolerant C4 cereals; (3) The transcriptional responses of *MGT* genes under Mg^2+^-, Ca^2+^-, or other divalent-cation-specific conditions remain insufficiently characterized in many crop species, while post-transcriptional regulation and associated signaling pathways under abiotic stresses (drought, salt, and low temperature) also remain largely unexplored. Given the strong correlation between Mg²^+^ utilization efficiency and crop stress tolerance/yield, elucidating the evolutionary patterns, functional diversity, and regulatory mechanisms of *MGT* genes is essential for understanding plant Mg²^+^ homeostasis and accelerating the development of stress-resilient cultivars.

Foxtail millet (*Setaria italica* L*.)*, a member of the Poaceae family, is one of the oldest domesticated cereals with a cultivation history of more than 8,000 years in northern China (Fukunaga and Kawase, 2024). It has strong adaptability to adverse environments (drought, barren soil and low temperature) and serves as a staple food in China, India and Africa (Ma et al., 2016). With its compact genome (~ 490 Mb), short life cycle (~ 90 days), self-pollinating habit and high drought tolerance, foxtail millet has become an ideal model for studying C4 photosynthesis and drought resistance mechanisms (Diao et al., 2014; Yang et al., 2020). Investigation of the *MGT* gene family in foxtail millet may provide important insights into Mg^2+^-related stress adaptation and identify candidate genes for future functional studies and molecular breeding, while also offering a useful reference for the functional characterization of *MGT* genes in other cereal crops. Here, we systematically identified and characterized the *SiMGT* gene family, aiming to reveal their evolutionary dynamics, expression patterns, and stress response mechanisms, and to provide candidate genes and molecular markers for genetic improvement of foxtail millet.

## 2 Materials and methods

### 2.1 Plant materials and different treatment

#### 2.1.1 Experimental site

The experiment was carried out at the Experimental Farm and Laboratory of Genetic Improvement for Special Crops, College of Agriculture, Henan University of Science and Technology (Luoyang, China; 34°61’N, 112°45’E).

#### 2.1.2 Experimental design

The foxtail millet cultivar ‘Jingu 5’ (a widely cultivated variety with high stress tolerance) was used as the plant material. The seeds were surface-sterilized in 75% ethanol for 30 s and 5% sodium hypochlorite for 5 min, washed five times with sterile water, and then sown in plastic pots (10 cm × 10 cm) containing clean moist fine sand. The pots were placed in a constant temperature incubator (28 °C, 16 h light/8 h dark photoperiod, 60% relative humidity) until seedlings reached the three-leaf stage. Uniform and healthy three-leaf-stage seedlings were then selected for four abiotic stress treatments: low temperature (4 °C), drought, salt (100 mmol/L NaCl), and ABA (100 mg/L). Each treatment included three biological replicates and untreated seedlings sampled at the corresponding time points served as controls. Seeds were used only for germination and seedling establishment, whereas all abiotic stress treatments were applied to three-leaf-stage seedlings.

Sampling procedures were as follows:

Drought treatment: Watering was withheld completely; seedlings were sampled immediately when wilting symptoms appeared (about 7 days after water withholding). The samples were snap-frozen in liquid nitrogen and stored at − 80 °C for subsequent analysis.

Low temperature treatment: Seedlings were transferred to a 4 °C incubator (16 h light/8 h dark), and samples were collected at 0, 3, 6, 12 and 24 h after initiation of treatment. At each time point, three plants with uniform growth were selected, rinsed with sterile water, separated into shoots and roots, snap frozen in liquid nitrogen and stored at − 80 °C.

Salt treatment: Seedlings were irrigated with NaCl solution (100 mmol/L, 50 mL per pot) and samples were collected at 0, 0.5, 1, 2, and 3 h after treatment. Three uniform plants were harvested at each time point, separated into shoots and roots, frozen in liquid nitrogen and stored at − 80 °C.

ABA treatment: Seedlings were sprayed with 100 mg/L ABA solution (containing 0.1% Tween-20) until the leaf surfaces were fully wetted, and samples were collected at 0, 0, 0.5, 1, 2, and 3 h after treatment. Three uniform plants were selected for each time point, separated into shoots and roots, frozen in liquid nitrogen and stored at −80 °C.

### 2.2 Methods

#### 2.2.1 Identification and sequence characterization of *SiMGT* genes

The foxtail millet genome sequence (v2.0), protein sequences, and GFF3 annotation file were downloaded from the MDSi multi-omics database (http://foxtail-millet.biocloud.net/home) (He et al., 2024). The AtMGT protein sequences of *Arabidopsis thaliana* were obtained from The *Arabidopsi*s Information Resource (TAIR, https://www.arabidopsis.org/), and the *OsMRS2* sequences of *Oryza sativa* were retrieved from Rice Annotation Project Database (RAP-DB, https://rapdb.dna.naro.go.jp/). These sequences were used as queries to perform BLASTP searches against the foxtail millet proteome with an E-value threshold of 1e-5. The hits were merged and filtered using TBtools v1.120 software (Chen et al., 2023) to create a preliminary dataset of candidate *SiMGT* genes.

The Hidden Markov Model (HMM) profile of the *MGT* family (Pfam ID: PF01544) was downloaded from the Pfam database (http://pfam.xfam.org/) (Mistry et al., 2021), and used to screen the foxtail millet proteome using HMMER 3.3.2 software (E-value ≤ 1e–10). The overlapping sequences identified by both BLASTP and HMMER analyses were selected as core candidates. To confirm the presence of a conserved *MGT* domain, candidate sequences were submitted to NCBI Conserved Domain Database (CDD, https://www.ncbi.nlm.nih.gov/Structure/bwrpsb/bwrpsb.cgi) (Letunic et al., 2021), SMART (http://smart.embl-heidelberg.de/) and InterPro (https://www.ebi.ac.uk/interpro/) for multilevel domain verification. Sequences lacking the characteristic CorA domain were excluded, and the final set of *SiMGT* family members was confirmed.

The physicochemical properties of SiMGT proteins, including the number of amino acids, molecular weight (MW), theoretical isoelectric point (pI), instability index, aliphatic index and grand average of hydropathicity (GRAVY) were predicted using ExPASy ProtParam online tool (https://web.expasy.org/protparam/) (Wilkins et al., 1999). Subcellular localization was predicted by Hum-mPLoc 2.0 online server (http://www.csbio.sjtu.edu.cn/bioinf/hum-multi-2/) (Horton et al., 2007) and WoLF PSORT (https://wolfpsort.hgc.jp/), and consistent results were taken as final predictions. The gene structure (exon–intron distribution) was analyzed by comparing coding sequences (CDS) with their corresponding genomic sequences using Gene Structure Display Server (GSDS, http://gsds.gao-lab.org/) and visualized using TBtools (Wilkins et al., 1999; Horton et al., 2007).

#### 2.2.2 Chromosomal localization, phylogenetic, and collinearity analysis

Chromosomal localization was performed using the “Gene Location Visualize” function in TBtools based on the foxtail millet genome annotation file (GFF3 format) and *SiMGT* gene sequences, and a chromosomal distribution map with a scale bar of 10 Mb was generated (Chao et al., 2021).

To explore the evolutionary relationships, protein sequences of *MGT* genes from six plant species (*Setaria italica*: 9 *SiMGTs*; *Arabidopsis thaliana*: 10 *AtMGTs*; *Oryza sativa*: 9 *OsMRS2s*; *Zea mays*: 12 *ZmMGTs*; *Sorghum bicolor*: 10 *SbMGTs*; *Setaria viridis*: 9 *SevirMGTs*) were aligned using ClustalX 2.1 with default parameters (gap opening penalty = 10, gap extension penalty = 0.2). The neighbor-joining (NJ) phylogenetic tree was constructed by MEGA 11 software with 10,000 bootstrap replicates, Poisson correction model and pairwise deletion of gaps (Kumar et al., 2018). The phylogenetic tree was further beautified and labelled using iTOL online platform (https://itol.embl.de/), and branches were colored according to different plant species.

Collinearity analysis was performed to explore the intra- and interspecies evolutionary relationships. The protein sequences of *MGT* genes from foxtail millet, *Arabidopsis thaliana*, *Oryza sativa*, and *Zea mays* were retrieved from Phytozome (https://phytozome-next.jgi.doe.gov/). Intraspecific (within foxtail millet) and interspecific (foxtail millet vs *A. thaliana*, foxtail millet vs *O. sativa*, foxtail millet vs *Z. mays*) collinearities were analyzed using the “Synteny Plot” function in TBtools with a minimum number of genes in collinear blocks set as 5. Syntenic relationship maps were generated with gray lines representing genome-wide collinear blocks and red lines indicating collinear *MGT* gene pairs (Subramanian et al., 2019).

#### 2.2.3 Protein structure and interacting protein prediction

Secondary structures of SiMGT proteins, including α-helix, β-sheet, random coil and extended strand were predicted using SOPMA online tool (https://npsa.lyon.inserm.fr/cgi-bin/npsa_automat.pl?page=/NPSA/npsa_sopma.html) with a confidence threshold of 80% (Combet et al., 2000). Tertiary structures were predicted by homology modeling using the SWISS-MODEL online platform (http://swissmodel.expasy.org/interactive) (Waterhouse et al., 2018). The highest-quality template was selected based on sequence identity (> 30%) and coverage (> 70%), and the generated models were validated by Ramachandran plot analysis using PROCHECK; models with more than 90% of residues in favored regions were considered reliable.

Taking the STRING web database (https://string-db.org/), a well-known online resource for protein–protein interaction research as an analytical basis, we developed a functional protein–protein interaction network model for MGT proteins with the confidence parameter uniformly set to 0.150.

#### 2.2.4 Cis-acting element and tissue-specific expression analysis

The 2000 bp promoter sequences (upstream of the transcription start site) of *SiMGT* genes were extracted from the foxtail millet genome using the “Extract Promoter” function in TBtools (Lescot, 2002). Cis-acting elements in the promoter regions were predicted using the PlantCARE online database (http://bioinformatics.psb.ugent.be/webtools/plantcare/html/) (Lescot, 2002) and PLACE (https://www.dna.affrc.go.jp/PLACE/), with redundant elements removed. The results were visualized with TBtools to show the types, numbers, and distribution positions of cis-acting elements, with different colors representing distinct element categories.

Tissue-specific expression data of *SiMGT* genes in roots, stems, leaves, and panicles (at the filling stage) were retrieved from the Ensembl Plants database (https://plants.ensembl.org/index.html) (Tang et al., 2023), with expression values represented as fragments per kilobase of transcript per million mapped reads (FPKM). The FPKM values were normalized using the z-score method, and a heatmap was generated using TBtools with the hierarchical clustering method (Euclidean distance), displaying tissue-specific expression patterns of *SiMGT* genes.

#### 2.2.5 Quantitative real-time PCR validation

Total RNA was extracted from foxtail millet seedling shoots and roots using the RNAiso Plus kit (TaKaRa, Dalian, China) according to the manufacturer’s instructions. RNA integrity was assessed by 1% agarose gel electrophoresis (two clear bands of 28S and 18S rRNA), and RNA concentration and purity were determined using a NanoDrop 2000 spectrophotometer (Thermo Fisher Scientific, Waltham, USA) with A260/A280 ratios between 1.8 and 2.0. First-strand cDNA was synthesized using the PrimeScript™ RT Reagent Kit with gDNA Eraser (TaKaRa, Dalian, China) to eliminate genomic DNA contamination. The synthesized cDNA was diluted 10-fold and stored at −20 °C for qRT-PCR analysis.

qRT-PCR was performed on a Bio-Rad LightCycler 96 Real-Time PCR System (Bio-Rad, Hercules, USA) using the TB Green^®^ Premix Ex Taq™ II kit (TaKaRa, Dalian, China). Specific primers for *SiMGT* genes and the internal reference gene SiActin were designed by Sangon Biotech (Shanghai, China) (**Table 1**), with primer specificity verified by melting curve analysis. The PCR reaction system (20 μL) included 10 μL of TB Green Premix Ex Taq II, 0.4 μL of forward primer (10 μmol/L), 0.4 μL of reverse primer (10 μmol/L), 2 μL of cDNA template, and 7.2 μL of sterile ddH_2_O. The PCR program was as follows: 95 °C for 30 s; 40 cycles of 95 °C for 5 s, 60 °C for 30 s; followed by melting curve analysis (65–95 °C, 0.5 °C increment per 5 s). Relative expression levels of target genes were calculated using the 2^(-ΔΔCT) method (Rao et al., 2013), with three biological replicates and three technical replicates per sample. Statistical analysis was performed using SPSS 26.0 software, and significant differences were determined by one-way ANOVA with Duncan’s multiple comparison test (P < 0.05). Graphs were generated using GraphPad Prism 8.0 software.

**Table 1:** Primers for qRT-PCR of *SiMGT* genes in foxtail millet.

Gene name	Forward primer (5’–3’)	Reverse primer (5’–3’)	Amplicon length (bp)
*SiMGT*1	AGACGCTGGAGATCGACAAGG	GTGGACGGGTAGACGAAGAGC	152
*SiMGT*2	GCATTCCAAGGATTCACCGAGAC	TCGTCGTCACTGTCTTCTTCCTC	187
*SiMGT*3	CTGCTGGAGTGGCTGTTGTTG	CGTTTGCCGCCCTTGTCTC	145
*SiMGT*4	GACGGAGCGGCAGAGGAC	ACTCGAACGGCAGGACCTTG	138
*SiMGT*5	GCCGCCTGCTCCTTCCTG	CGTTCCAAGTTGAGGGTGCTG	163
*SiMGT*6	TGCTGGACGCCGACAAGTAC	CGCCCGAGGATGGTGGAAG	129
*SiMGT*7	TGCCGCTGCCGCTTCTG	TCCTCTTCCTCCTCCATCACCTC	176
*SiMGT*8	TTCTCCCACTCCTCAAGCATCC	AGCACCTCCTCGGCAGTAAC	158
*SiMGT*9	TCCTGCTGCTGGAACCTCTTG	GCGTGGCACCAACATCAACC	141
*SiActin*	GGTGCCCTGAGGTGCTGTTC	ACCACTGAGGACGACATTACCATAC	167

#### 2.2.6 Superior haplotype analysis

Genotype data of foxtail millet *MGT* genes and phenotypic data of 1844 foxtail millet accessions were downloaded from the Setaria DB database (https://111.203.21.71:8000/index.html). Coding sequences of *SiMGT* genes were used as target regions, and single nucleotide polymorphisms (SNPs) with a minimum allele frequency (MAF) ≥ 0.05 were retained to exclude low-frequency alleles (Zuo et al., 2023). Phenotypic data included panicle length, panicle diameter, and panicle weight from three environments: Changzhi (Shanxi Province, 2011), Anyang (Henan Province, 2016), and Shunyi (Beijing, 2011). Association analysis between SNPs and phenotypic traits was performed using the mixed linear model (MLM) in TASSEL 5.0 software, with population structure (Q matrix) and kinship (K matrix) as covariates to control false positives. Haplotypes were constructed based on significant SNPs (P < 0.01), and superior haplotypes associated with favorable yield traits were identified by comparing phenotypic differences among haplotypes using Student’s t-test (P < 0.05).

#### 2.2.7 Data processing and analysis

All collected data were subjected to statistical analysis and visualization via GraphPad Prism 8.0 software (San Diego, CA, USA). For each group, three replicates were established. Notably, significant differences between data sets were represented by ‘*’ for values where 0.01 < p ≤ 0.05, or by ‘**’ for those with p ≤ 0.01, as determined through the Student’s t-test.

## 3 Results

### 3.1 Genome-wide identification and physicochemical properties of *SiMGT* genes

A total of nine *SiMGT* genes (*SiMGT1*–*SiMGT9*) were identified in foxtail millet via combined HMM and BLASTP screening, followed by multi-level conserved domain validation (CDD, SMART, InterPro). The amino acid lengths of SiMGT proteins range from 386 aa (*SiMGT*6) to 532 aa (*SiMGT*1), with corresponding molecular weights of 42.70 kDa (*SiMGT*6) to 59.74 kDa (*SiMGT*1) (**Table 2**). The theoretical isoelectric points (pI) of SiMGT proteins vary from 4.74 (*SiMGT*4) to 8.88 (*SiMGT*9), with *SiMGT*1 (pI = 7.64) and *SiMGT*9 classified as basic proteins (pI > 7), and the remaining seven members as acidic proteins (pI < 7). The instability indices of SiMGT proteins range from 40.18 (*SiMGT*6) to 60.71 (*SiMGT*7), with *SiMGT*7 identified as unstable (instability index > 60) and the others as relatively stable (instability index 40–60). All SiMGT proteins exhibit negative GRAVY values (−0.294 to −0.032), indicating hydrophilic properties, with *SiMGT*2 showing the strongest hydrophilicity (GRAVY = −0.294) and *SiMGT*9 the weakest (GRAVY = −0.032).

**Table 2:** Physicochemical properties and subcellular localization of *SiMGT* proteins.

Protein ID	Gene name	Amino acid number (aa)	Molecular weight (KDa)	Isoelectric point (pl)	Instability index	Hydropathicity(GRAVY)	Subcellular localization
KQL11173	*SiMGT1*	532	59.74	7.64	53.88	-0.154	Cytoplasm. Mitochondrion. Nucleus.
KQL07898	*SiMGT2*	410	45.64	5.00	42.24	-0.294	Nucleus.
KQL08231	*SiMGT3*	442	48.21	4.78	46.20	-0.225	Chloroplast. Nucleus.
KQK97282	*SiMGT4*	411	44.03	4.74	45.96	-0.180	Chloroplast. Cytoplasm. Mitochondrion. Nucleus. Peroxisome.
KQK97945	*SiMGT5*	434	48.13	5.34	49.81	-0.061	Nucleus.
KQK87164	*SiMGT6*	386	42.70	4.86	40.18	-0.198	Chloroplast. Nucleus.
KQK87700	*SiMGT7*	429	47.07	5.11	60.71	-0.05	Nucleus.
KQK90101	*SiMGT8*	479	52.23	6.29	52.12	-0.14	Chloroplast.
KQK92670	*SiMGT9*	435	48.59	8.88	43	-0.032	Nucleus.

Subcellular localization prediction reveals diverse distribution patterns of SiMGT proteins: *SiMGT*1 was predicted to localize to the cytoplasm, mitochondria, and nucleus; *SiMGT*4 was predicted to be distributed in the chloroplast, cytoplasm, mitochondria, nucleus, and peroxisome; *SiMGT*3, *SiMGT*6, and *SiMGT*8 were mainly predicted to localize to the chloroplast and nucleus (*SiMGT*8 exclusively in chloroplast); and *SiMGT*2, *SiMGT*5, *SiMGT*7, and *SiMGT*9 were predominantly predicted to localize to the nucleus (**Table 2**). These results suggest that *SiMGT*s may mediate Mg²^+^ transport across multiple subcellular compartments, and may therefore participate in diverse physiological processes.

### 3.2 Phylogenetic analysis of *SiMGT* genes

**Figure 1:**
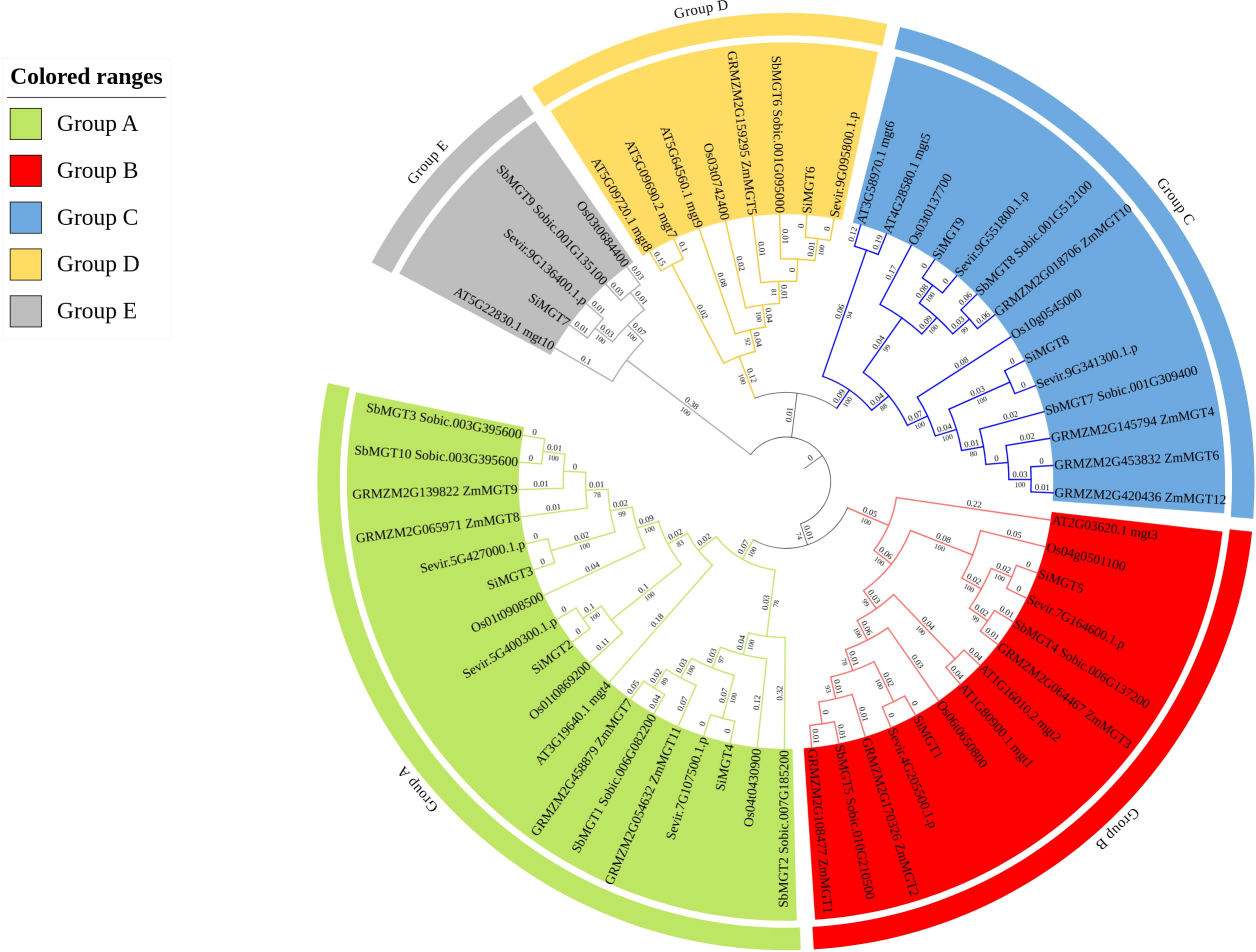
Phylogenetic tree of MGT proteins from *Setaria italica, Arabidopsis thaliana, Oryza sativa, Sorghum bicolor, Zea mays, and Setaria viridis*. *Si, Setaria italica; At, Arabidopsis thaliana; Os, Oryza sativa; Sb, Sorghum bicolor; Zm, Zea mays; Sevir, Setaria viridis*. Bootstrap values (10,000 replicates) are indicated at branch nodes, and different colors represent different plant species. The scale bar represents the number of amino acid substitutions per site.

The phylogenetic tree constructed with 59 MGT protein sequences from six plant species clusters *SiMGTs* into five distinct groups (Group A–E) (**Figure 1**). Group A includes *SiMGT2*, *SiMGT3*, and *SiMGT4*; Group B contains *SiMGT1* and *SiMGT5*; Group C comprises *SiMGT8* and *SiMGT9*; Group D and Group E each contain a single member (*SiMGT6* and *SiMGT7*, respectively). The presence of single-member groups (D and E) suggests that these genes may have undergone unique functional divergence during evolution or retained ancestral sequence characteristics.

*SiMGT* members in Groups A, B, and C show close clustering with *MGT* homologs from graminaceous crops (rice, maize, sorghum), with bootstrap values >80%, indicating high evolutionary conservation of the *MGT* gene family within the Poaceae family. For example, *SiMGT2* clusters with *OsMRS2-3* and *ZmMGT4*, and *SiMGT8* clusters with *SbMGT8* and *SevirMGT8*, suggesting functional conservation among these homologs. Additionally, partial *SiMGT* members (e.g., *SiMGT1*) cluster with *AtMGT* genes from *Arabidopsis thaliana*, reflecting the ancient origin of the *MGT* gene family in angiosperms and conservation of core functional domains across monocots and dicots.

### 3.3 Chromosomal localization and collinearity analysis

**Figure 2:**
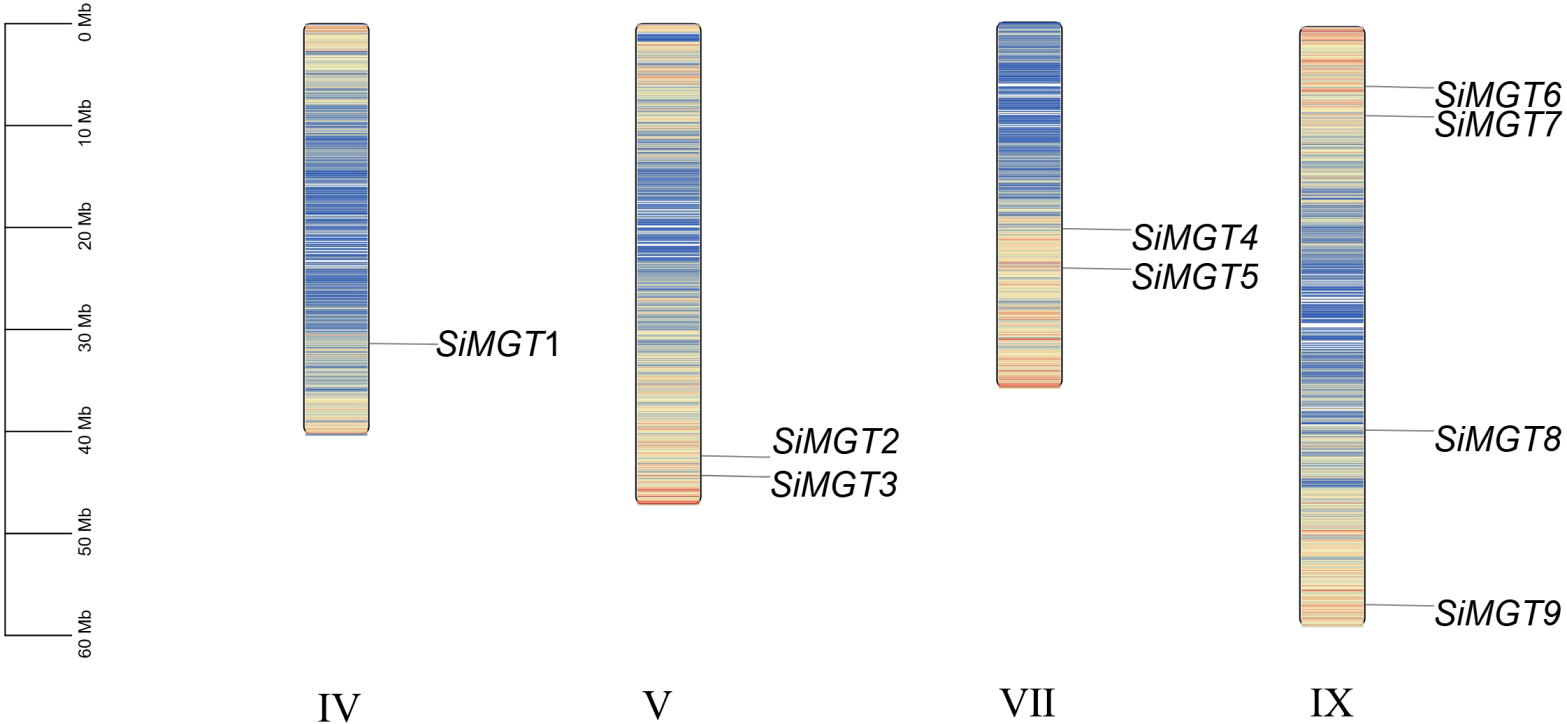
Chromosomal localization of *SiMGT* gene family members. Chromosome numbers are indicated at the top of each chromosome, and the scale bar represents 10 Mb. The gene name is labeled below the chromosome, and the length of the chromosome is proportional to its actual physical length.

Chromosomal localization analysis shows that the nine *SiMGT* genes are unevenly distributed across four of the nine foxtail millet chromosomes (**Figure 2**). Chromosome 9 harbors the highest number of *SiMGT* genes (4 members: *SiMGT*6, *SiMGT*7, *SiMGT*8, *SiMGT*9), followed by Chromosomes 5 and 7 (2 members each: *SiMGT*2 and *SiMGT*3 on Chromosome 5; *SiMGT*4 and *SiMGT*5 on Chromosome 7), and Chromosome 4 (1 member: *SiMGT*1). No *SiMGT* genes are detected on Chromosomes 1, 2, 3, 6, or 8, exhibiting a “localized clustering” distribution pattern, which may facilitate functional coordination among family members.

**Figure 3:**
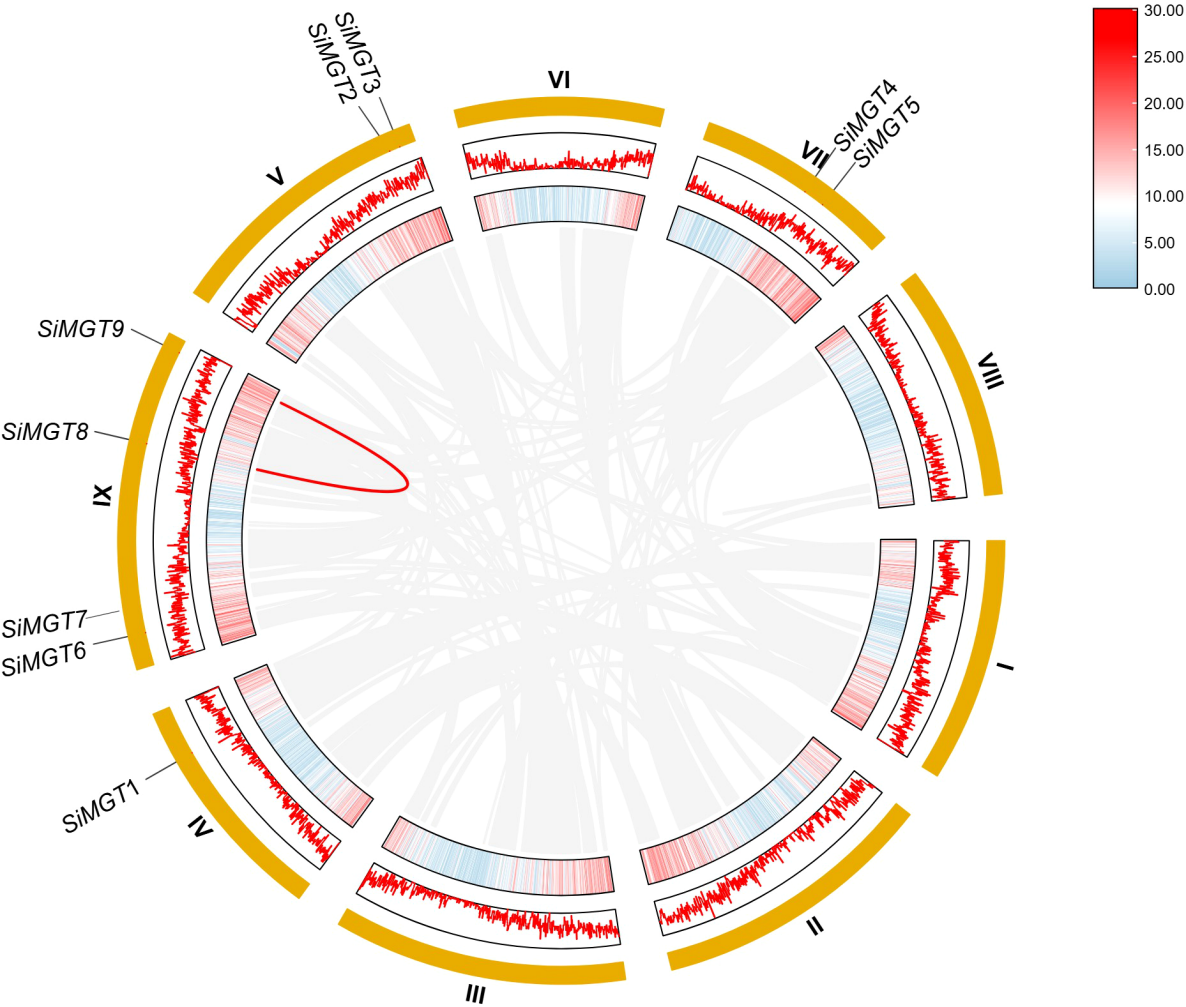
Intraspecific collinearity of *SiMGT* genes in foxtail millet. Gray lines represent collinear blocks across the genome, and red lines indicate the collinear *SiMGT* gene pair (*SiMGT*8/*SiMGT*9). The chromosome number is labeled at the top and left of the figure.

**Figure 4:**
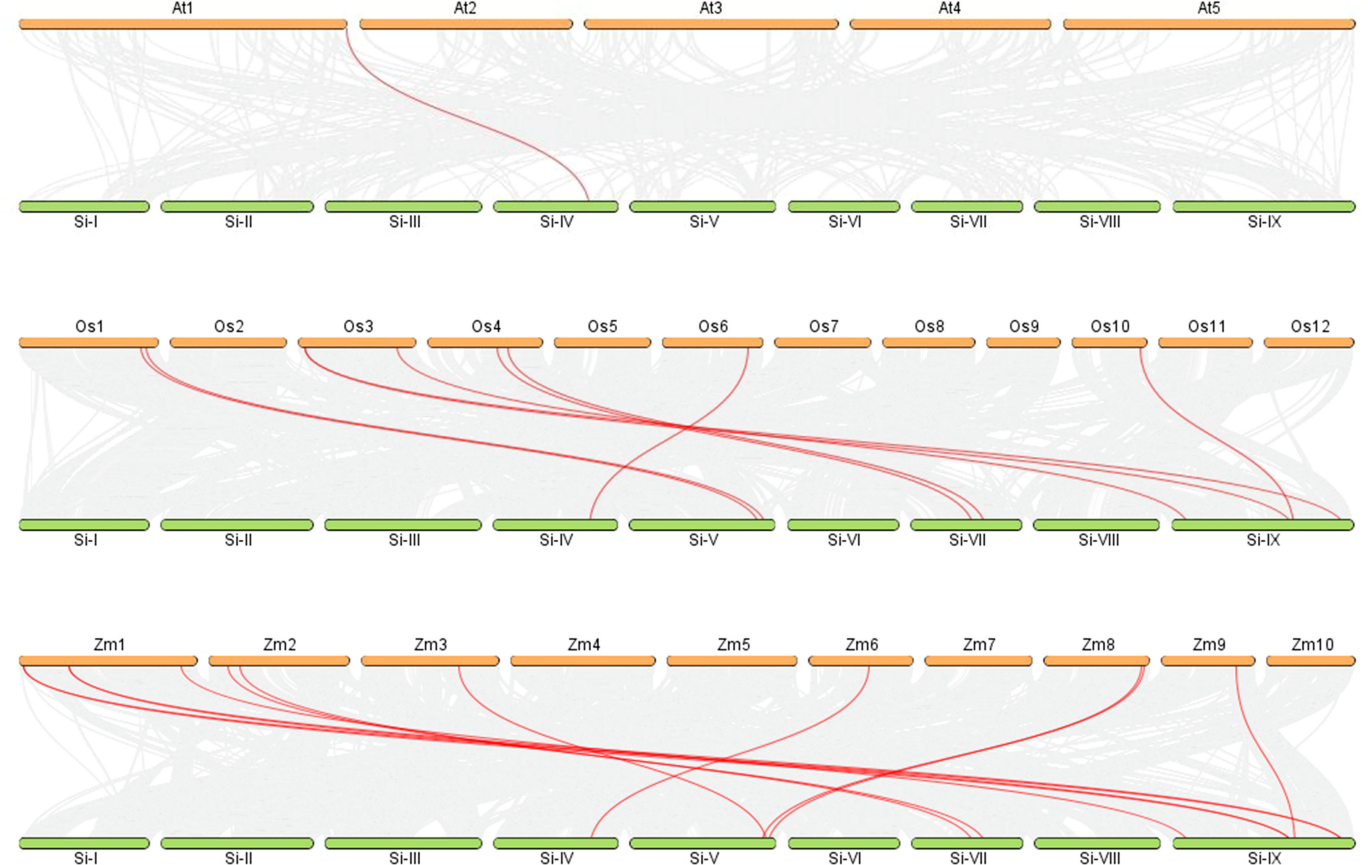
Interspecific collinearity of *MGT* genes between *Setaria italica*, *Arabidopsis thaliana*, *Oryza sativa*, and *Zea mays*. Gray lines represent genome-wide collinear blocks, and red lines indicate collinear *MGT* gene pairs between species. Chromosome numbers of each species are labeled at the top and left of the figure.

Intraspecific collinearity analysis identifies a single segmental duplication event between *SiMGT*8 and *SiMGT*9 (**Figure 3**), with a synonymous substitution rate (Ks) of 0.32, indicating that this duplication event occurred approximately 16 million years ago (assuming a Ks substitution rate of 2e-8 per site per year). No tandem duplication events are detected among other *SiMGT* members, indicating that the expansion of the *SiMGT* gene family in foxtail millet is primarily driven by limited segmental duplication rather than large-scale genome duplication. Interspecific collinearity analysis reveals 10 homologous gene pairs between foxtail millet and maize, 8 pairs between foxtail millet and rice, and only 1 pair between foxtail millet and Arabidopsis thaliana (**Figure 4**), confirming closer evolutionary relationships among graminaceous crops and supporting the monocot-specific conservation of *MGT* genes.

### 3.4 Gene structure and protein motif analysis

**Figure 5:**
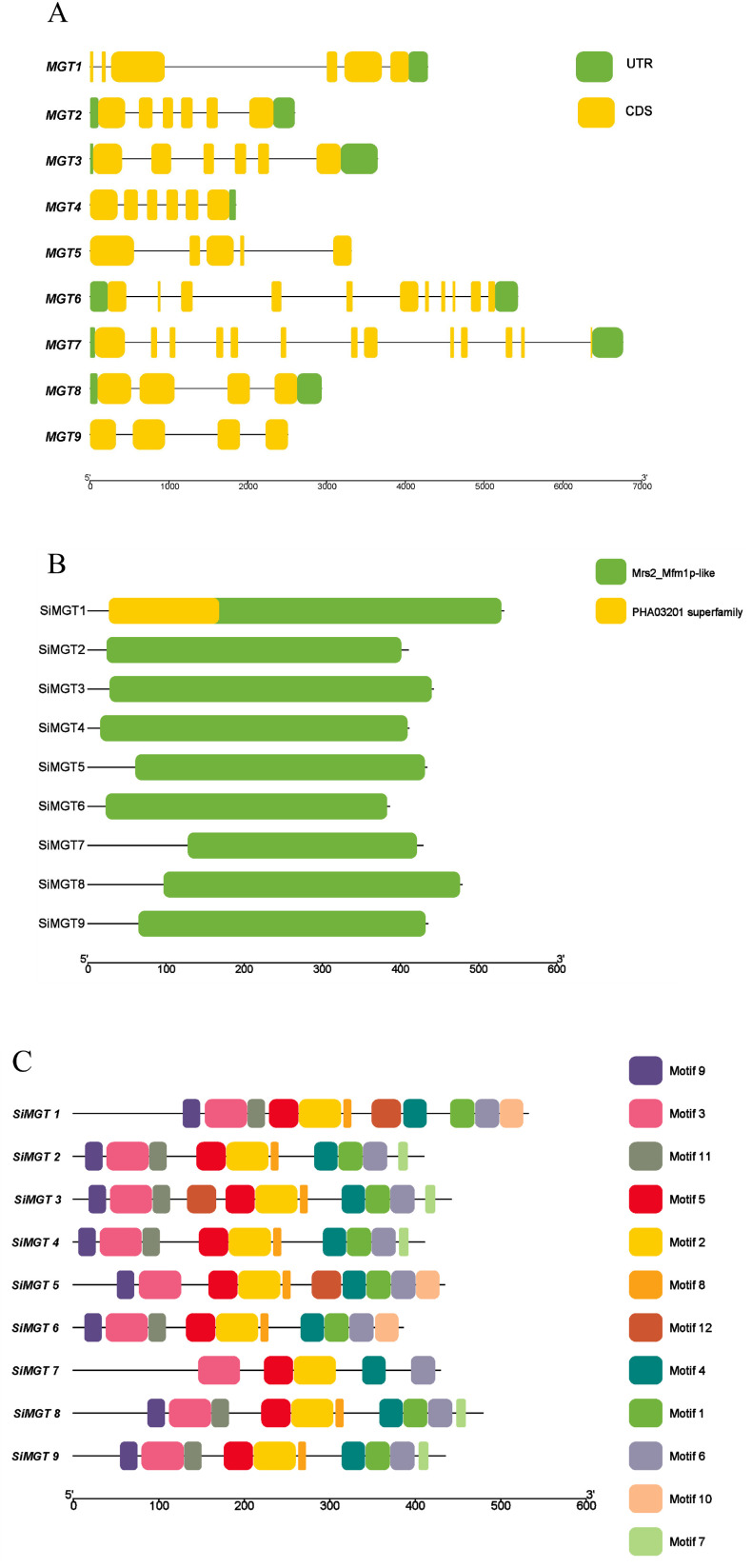
**(A)** Gene structures of *SiMGT* genes. Green boxes represent coding sequences (CDS), yellow boxes represent untranslated regions (UTRs), and black lines represent introns. The scale bar indicates sequence length (bp). **(B)** Functional domain architecture of SiMGT proteins in foxtail millet. Green boxes represent the conserved Mrs2_Mfm1p-like domain, and yellow boxes represent the PHA03201 superfamily domain. Black lines indicate the full-length protein sequences, and the scale bar indicates protein length (aa). **(C)** Conserved motifs of SiMGT proteins. Different colors represent distinct motifs (Motif 1–12), and the scale bar indicates sequence length (aa).

Gene structure analysis shows that most *SiMGT* genes have relatively conserved CDS lengths and short untranslated regions (UTRs), with 2–5 exons (**Figure 5A**). Notably, *SiMGT*7 exhibits a longer gene structure (7 exons) with more exons/introns compared to other members (2–4 exons), suggesting structural expansion and functional differentiation during evolution, which may contribute to its specific stress response. Functional domain analysis showed that all SiMGT proteins contained the conserved Mrs2_Mfm1p-like domain, whereas SiMGT1 additionally carried a PHA03201 superfamily annotation in the N-terminal region (**Figure 5B**). Conserved motif analysis identifies 12 distinct conserved motifs (Motif 1–12) in SiMGT proteins using the MEME online tool (**Figure 5C**). Members of the same phylogenetic group (e.g., SiMGT2, SiMGT3, SiMGT4) share identical motif types and arrangement patterns, indicating high sequence conservation and potential functional similarity within evolutionary clades. SiMGT7 contains only five core motifs (Motif 3, 5, 2, 4, 6), suggesting motif loss during evolution, which may be associated with functional specialization. In contrast, SiMGT1 harbors all 12 conserved motifs, implying a more comprehensive functional role in Mg²^+^ transport and regulation.

### 3.5 Secondary and tertiary structure analysis

Secondary structure prediction shows that α-helices are the most abundant structural element in SiMGT proteins (43.59–60.88%), followed by random coils (32.12–48.95%), extended strands (5.85–7.82%), and β-sheets (0.00–0.01%) (**Table 3**). The high proportion of α-helices (average 52.63%) suggests a transmembrane structural feature, which is consistent with the transport function of SiMGT proteins. For example, *SiMGT*6 has the highest α-helix content (60.88%), implying a more stable transmembrane structure.

**Table 3:** Secondary structure composition of *SiMGT* proteins.

Gene name	α-helix (%)	Extended strand (%)	β-sheet (%)	Random coil (%)	Distribution of secondary structure elements	Three-dimensional structure	Tertiary structure reliability (favored region, %)
SiMGT1	0.4492	0.062	0.01	0.4887	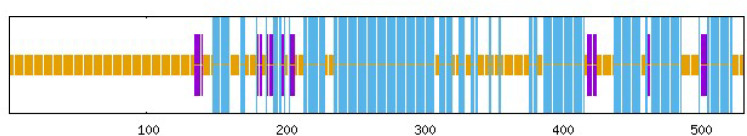	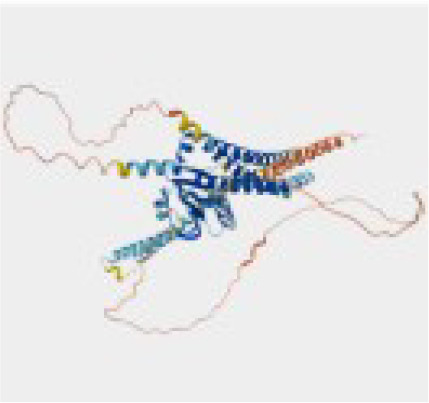	92.3
SiMGT2	0.5805	0.0659	0.00	0.3537	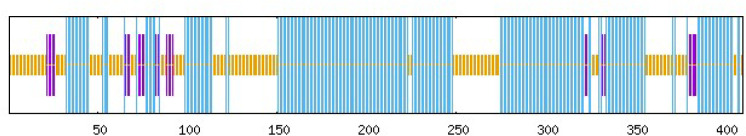	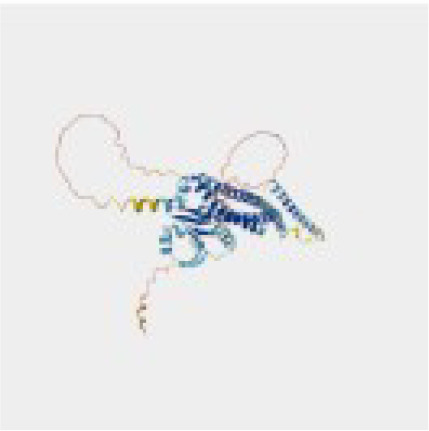	93.1
SiMGT3	0.5973	0.0611	0.00	0.3416	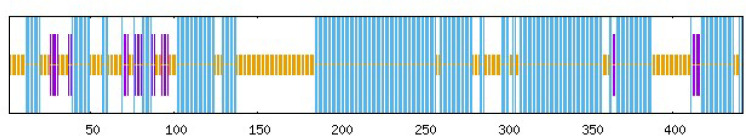	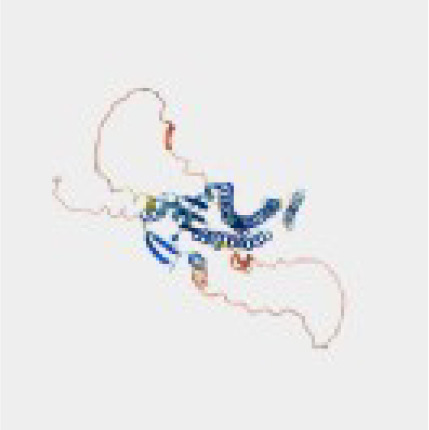	91.8
SiMGT4	0.5547	0.0754	0.01	0.3698	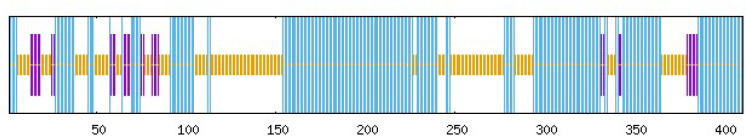	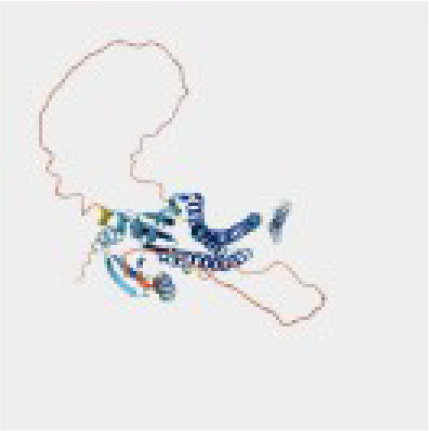	92.7
SiMGT5	0.5184	0.0622	0.00	0.4194	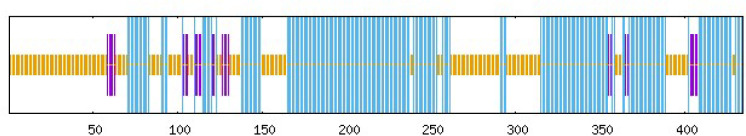	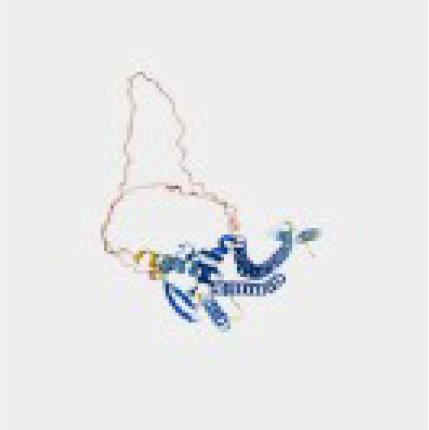	90.5
SiMGT6	0.6088	0.0699	0.01	0.3212	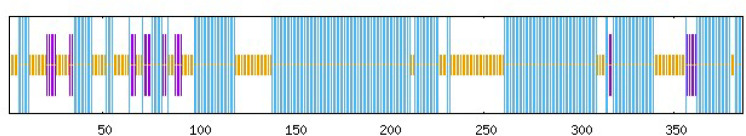	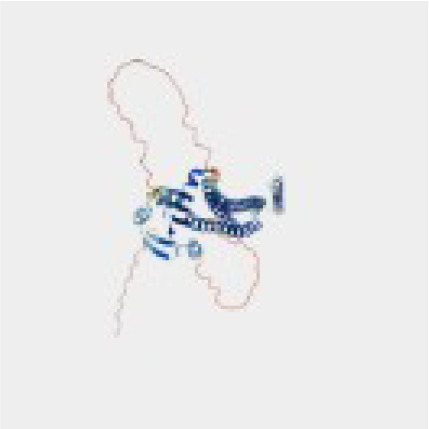	94.2
SiMGT7	0.4359	0.0746	0.00	0.4895	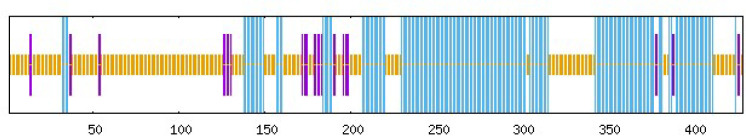	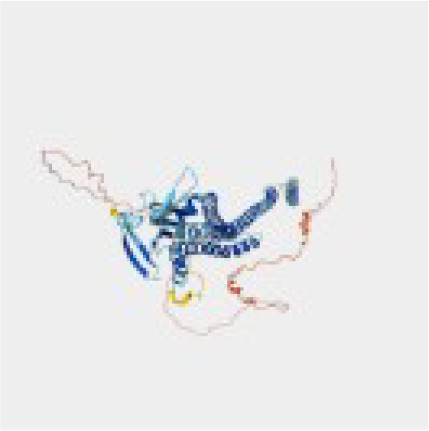	91.2
SiMGT8	0.4635	0.0585	0.00	0.4781	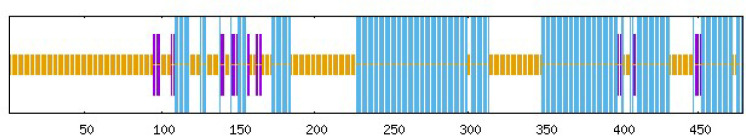	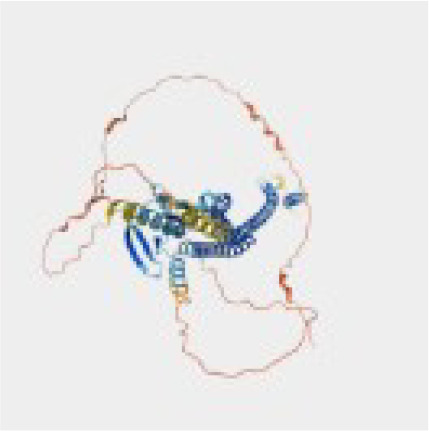	92.5
SiMGT9	0.5011	0.0782	0.00	0.4207	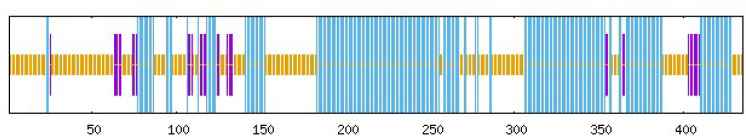	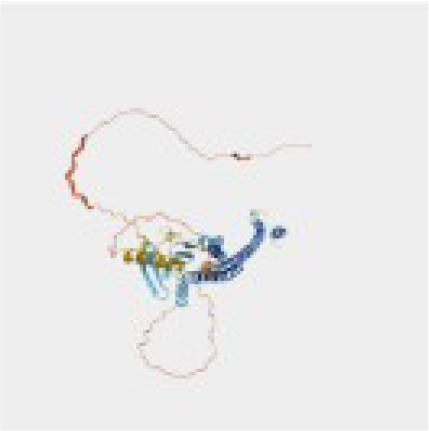	93,4

Tertiary structure modeling reveals conserved folding patterns among SiMGT proteins, with the core region consisting of multiple α-helices and loops (**Table 3**). Structural differences are primarily observed in loop regions, which may contribute to functional divergence by mediating protein-protein interactions or substrate binding specificity. Ramachandran plot validation shows that >90% of residues in all *SiMGT* tertiary models are in the favored region, indicating high model reliability.

### 3.6 Prediction and correlation analysis of interacting proteins

To elucidate the potential functional coordination among *SiMGT* members, a predictive protein-protein interaction (PPI) network was constructed. Due to the limited experimental interaction data specifically for Setaria italica in the current STRING database, applying a medium confidence threshold directly to *SiMGTs* yielded no connections. To overcome this database limitation and provide a biologically rigorous model, the nine SiMGT proteins were mapped to their well-annotated orthologs in the model plant Arabidopsis thaliana.

**Figure 6:**
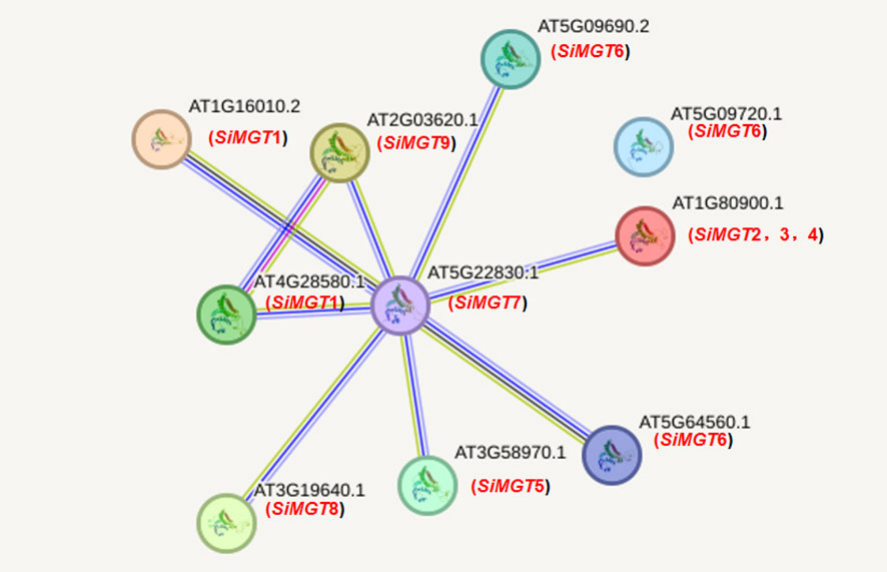
Predictive protein-protein interaction (PPI) network of *SiMGT* family members based on *Arabidopsis thaliana* orthologs. Due to the limited experimental interaction data for *Setaria italica* in the current database, *SiMGT* proteins were mapped to their corresponding Arabidopsis orthologs to construct a robust functional network. The network was generated using the STRING database with a medium confidence threshold of 0.400. Node labels indicate the Arabidopsis gene IDs, with the corresponding *SiMGT* members provided in red parentheses. Edges represent protein-protein associations, and lines of different colors indicate distinct types of interaction evidence.

Utilizing a robust medium confidence threshold of 0.400, the ortholog-based network (**Figure 6**) revealed a highly interconnected hub-and-spoke topology. The network features a central hub corresponding to AT5G22830.1 (the ortholog of *SiMGT7*), which interacts directly with multiple peripheral nodes. Furthermore, additional specific associations among peripheral nodes were observed, such as the interactions among *AT4G28580.1 (SiMGT1*), *AT2G03620.1* (*SiMGT9*), and *AT5G09690.2* (*SiMGT6*).

Given the high evolutionary conservation of the *MGT* family, this orthology-based network reliably reflects the potential, functionally conserved interaction patterns of *SiMGT* proteins. These topological characteristics suggest that *SiMGT7* likely serves as a core regulatory hub, playing a crucial role in mediating metabolic pathways and cellular signal transduction, while the remaining members form functional modules to synergistically participate in physiological activities.

### 3.7 Cis-acting element analysis

**Figure 7:**
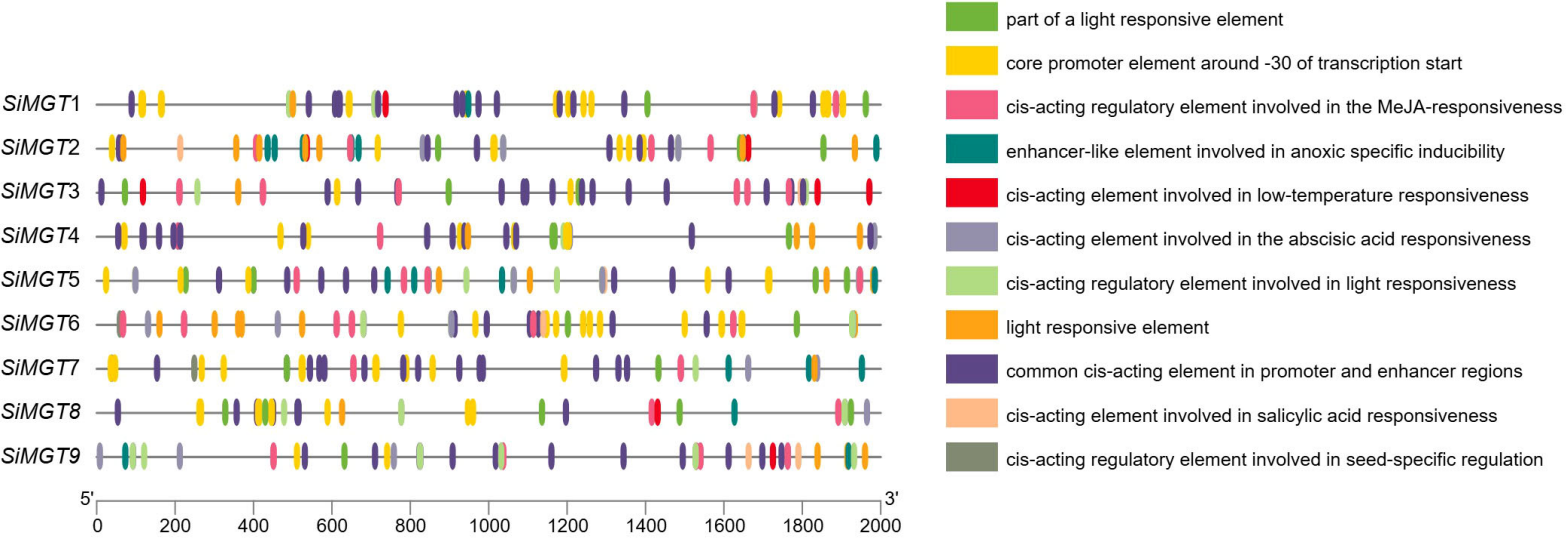
Distribution of cis-acting elements in the 2000 bp promoter regions of *SiMGT* genes. Different colors represent distinct types of cis-acting elements (light-responsive, stress-responsive, hormone-responsive). The scale bar indicates the length of the promoter region (bp), with the transcription start site at position 0.

Cis-acting element prediction identifies three major classes of elements in *SiMGT* promoters: light-responsive elements, stress-responsive elements, and hormone-responsive elements (**Figure 7**). Light-responsive elements (e.g., G-box, AT1-motif, GT1-motif) are present in all *SiMGT* promoters, with an average of 4–6 elements per promoter, suggesting that *SiMGT* genes may be associated with light-responsive regulation and potentially with photosynthesis-related processes. Stress-responsive elements include low-temperature-responsive elements (LTR), drought-responsive elements (MBS), and salt-responsive elements (TC-rich repeats): seven *SiMGT*s contain MBS, four contain LTR, and three contain TC-rich repeats, indicating their potential involvement in drought, cold, and salt stress adaptation.

Hormone-responsive elements include abscisic acid (ABA)-responsive elements (ABRE), auxin-responsive elements (TGA-element), and gibberellin-responsive elements (GARE-motif), which are distributed in all *SiMGT* promoters. Specifically, all nine *SiMGT*s contain ABRE, supporting a potential association between *SiMGT* gene expression and ABA-mediated regulation. Additionally, six *SiMGT*s contain TGA-element, and four contain GARE-motif, indicating regulation by auxin and gibberellin, respectively. These results suggest that *SiMGT* genes integrate multiple hormonal and environmental signals to regulate Mg²^+^ transport during plant growth and stress response.

### 3.8 Tissue-specific expression patterns

**Figure 8:**
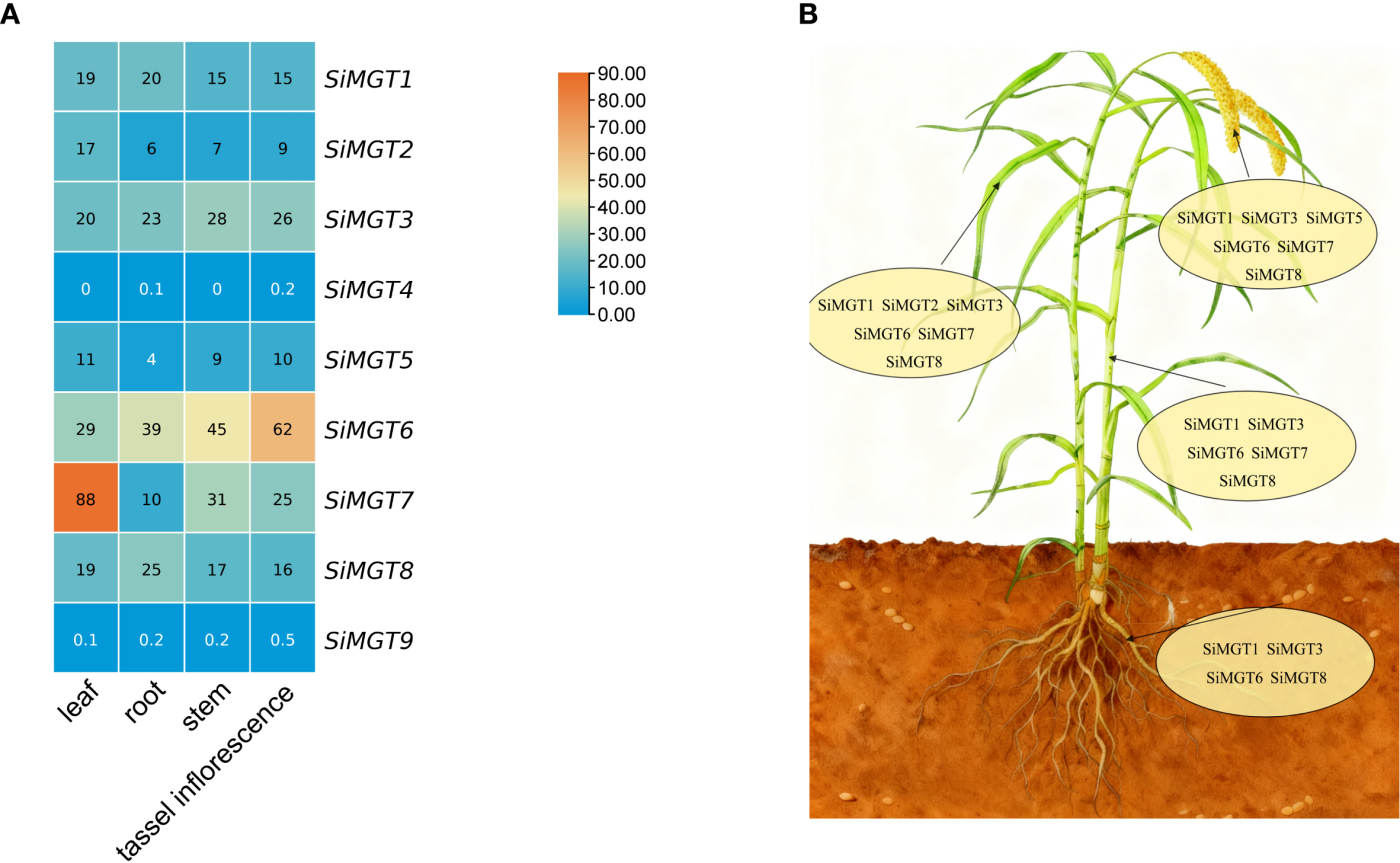
Tissue-specific expression patterns of *SiMGT* genes. **(A)** Heatmap of *SiMGT* gene expression in roots, stems, leaves, and panicles. Color intensity represents normalized expression levels (z-score of FPKM). **(B)** Expression levels of *SiMGT* genes in different tissues. Data are presented as mean ± SD of three biological replicates.

Tissue-specific expression profiling reveals distinct expression patterns of *SiMGT* genes across roots, stems, leaves, and panicles (**Figure 8**). *SiMGT*1, *SiMGT*3, *SiMGT*6, *SiMGT*7, and *SiMGT*8 are constitutively highly expressed in all four tissues (FPKM > 10), suggesting they are core regulators of basal Mg²^+^ homeostasis in foxtail millet. In contrast, *SiMGT*4 and *SiMGT*9 show relatively low expression levels (FPKM < 5) across all tissues, implying they may be induced under specific developmental stages or stress conditions.

Notably, *SiMGT*7 exhibits leaf-specific high expression (FPKM = 89.6), approximately 90-fold higher than other tissues, suggesting a potential role in leaf-related Mg²^+^ transport or photosynthesis-associated processes.*SiMGT*6 shows panicle-preferential expression (FPKM = 42.3), with secondary enrichment in stems (FPKM = 28.7), suggesting involvement in Mg²^+^ allocation during panicle development and pollen formation. *SiMGT*3 is moderately expressed in stems (FPKM = 35.6) and panicles (FPKM = 31.2), characterizing it as a stem- and reproductive organ-preferential gene, potentially contributing to stem structural metabolism and reproductive development. These tissue-specific expression patterns reflect functional division of labor within the *SiMGT* family, ensuring precise regulation of Mg²^+^ transport in different tissues.

### 3.9 Expression responses to abiotic stresses

#### 3.9.1 Drought stress

**Figure 9:**
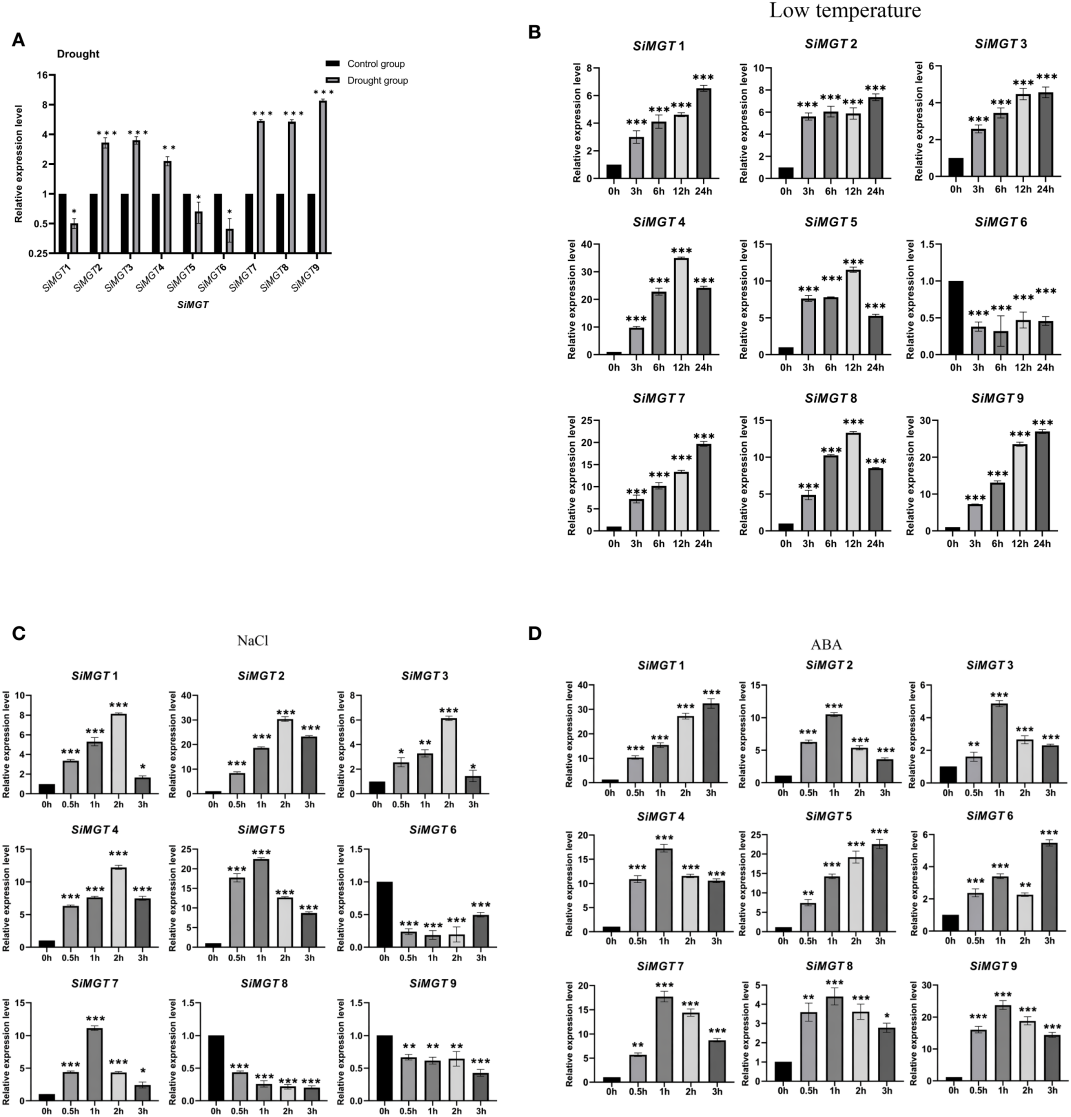
Expression patterns of *SiMGT* genes under abiotic stresses. **(A)** Drought stress; **(B)** Low-temperature stress; **(C)** Salt stress; **(D)** ABA stress. Relative expression levels are normalized to the control (0 h/min). Data are presented as mean ± SD of three biological replicates. *P < 0.05, **P < 0.01, ***P < 0.001 (one-way ANOVA with Duncan’s multiple comparison test).

Under drought stress, *SiMGT*2, *SiMGT*3, *SiMGT*4, *SiMGT*7, *SiMGT*8, and *SiMGT*9 are significantly upregulated in shoots and roots, with *SiMGT*9 showing the highest expression level (approximately 8-fold higher than the control) in shoots, followed by *SiMGT*7 and *SiMGT*8 (approximately 5-fold higher) (**Figure 9**). These expression patterns suggest that these genes may be involved in drought-responsive regulation. In contrast, *SiMGT*1, *SiMGT*5, and *SiMGT*6 are downregulated in both shoots and roots, indicating that different SiMGT members may exhibit divergent transcriptional responses under drought stress.

#### 3.9.2 Low-temperature stress

During low-temperature treatment (0–24 h), *SiMGT*4 reaches its expression peak at 12 h (approximately 35-fold higher than the control) in shoots, while *SiMGT*7 and *SiMGT*9 peak at 24 h (approximately 20-fold and 25-fold higher, respectively) (**Figure 9**). These three genes show sustained upregulation throughout the treatment period in both shoots and roots, suggesting that they may participate in cold-responsive regulation. *SiMGT*1, *SiMGT*2, and *SiMGT*3 exhibit gradual upregulation from 3 h to 12 h and maintain high expression levels at 24 h (e.g., *SiMGT*2: approximately 7-fold higher in shoots), indicating that multiple SiMGT members may respond coordinately to low-temperature treatment. *SiMGT*6 is consistently downregulated under low-temperature stress in both tissues.

#### 3.9.3 Salt stress

Under salt stress (100 mmol/L NaCl), *SiMGT*2 shows the most prominent response, with expression peaking at 2 h (approximately 30-fold higher than the control) in shoots (**Figure 9**). *SiMGT*1 and *SiMGT*4 peak at 2 h and remain significantly higher than the control in both shoots and roots, suggesting they mitigate sodium ion toxicity by enhancing Mg²^+^ transport during the early stages of salt stress. *SiMGT*6, *SiMGT*8, and *SiMGT*9 show relatively low expression levels in shoots and roots, but *SiMGT*6 is significantly upregulated at 180 min (approximately 3-fold higher) in roots, implying a specialized role in the late stages of salt stress response in roots.

#### 3.9.4 ABA stress

Under ABA treatment, *SiMGT*1, *SiMGT*2, and *SiMGT*3 rapidly reach expression peaks within 0.5-1 h (e.g., *SiMGT*2: approximately 10-fold higher at 1 h) in shoots, followed by gradual decline (**Figure 9**). These genes are likely involved in early ABA signal transduction, providing rapid regulation for stress response initiation. *SiMGT*4, *SiMGT*5, and *SiMGT*7 maintain high expression levels after peaking at 1 h (e.g., *SiMGT*4: >10-fold higher at 2 and 3 h) in both shoots and roots, while *SiMGT*9 shows stable expression after 1 h, suggesting they contribute to long-term ABA-mediated stress tolerance by sustaining Mg²^+^ homeostasis.

### 3.10 Superior haplotype analysis

**Figure 10:**
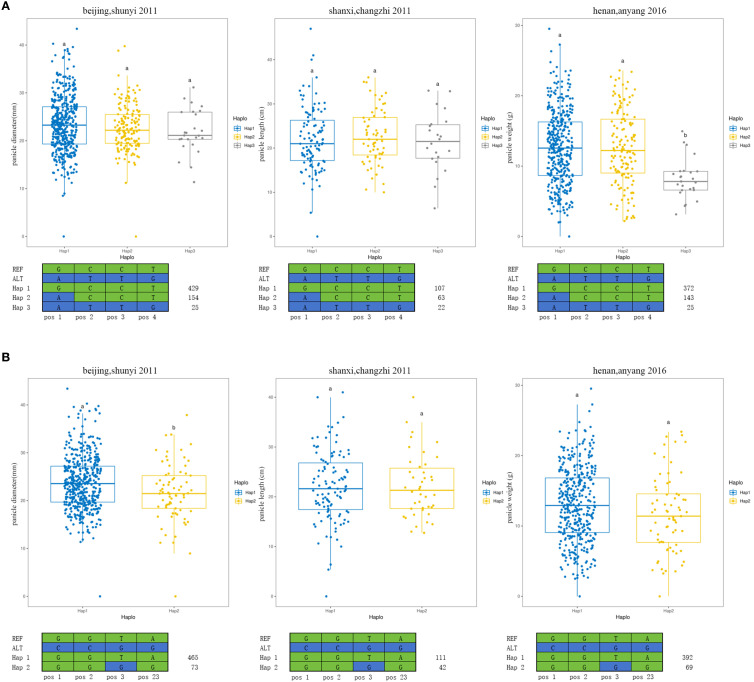
Superior haplotypes of *SiMGT*6 and *SiMGT*7 associated with yield traits and stress resistance. **(A)** Haplotypes of *SiMGT*6 and their association with panicle length, panicle diameter, and panicle weight. **(B)** Haplotypes of *SiMGT*7 and their association with drought tolerance index and cold tolerance index. Different letters indicate significant differences (P < 0.05, student’s t-test).

Given the tissue-specific and stress-responsive expression patterns of *SiMGT*6 and *SiMGT*7, haplotype analysis was performed for these two genes. Three haplotypes were identified for *SiMGT*6 (Hap1, Hap2, Hap3) based on two significant SNPs (SNP1: C/T, SNP2: A/G) in the coding sequence, and two haplotypes for *SiMGT*7 (Hap1, Hap2) based on one significant SNP (SNP3: G/A) (**Figure 10**). Association analysis with yield-related traits across three environments shows that *SiMGT*6 Hap1 (SNP1=C, SNP2=A) is the superior haplotype, with significantly higher panicle length (18.7 ± 1.2 cm), panicle diameter (5.3 ± 0.4 mm), and panicle weight (2.8 ± 0.3 g) compared to Hap2 and Hap3 (P < 0.05). For SiMGT7, Hap1 (SNP3 = G) was identified as the superior haplotype based on the significant phenotypic differences between haplotypes in the analyzed dataset. These superior haplotypes provide valuable molecular markers for foxtail millet breeding.

## 4 Discussion

Foxtail millet, as a drought-tolerant C4 cereal and model plant for stress resistance research, provides an excellent system to dissect the potential roles of Mg²^+^ transporter genes in abiotic stress adaptation. In this study, nine *SiMGT* genes were identified in foxtail millet, a number consistent with that in rice (9 *OsMRS*2 genes) (Saito et al., 2013) and Arabidopsis (10 *AtMGT* genes) (Li et al., 2001), reflecting the evolutionary conservation of the *MGT* gene family across plant species. Phylogenetic analysis clustered *SiMGT*s into five groups, with Groups A–C showing close affinity with graminaceous crop homologs, highlighting functional conservation within the Poaceae family, while Groups D and E (single-member groups) suggest lineage-specific functional divergence, potentially adapting to foxtail millet’s unique stress tolerance traits (Diao et al., 2014; Yang et al., 2020). For example, *SiMGT*7 (Group E) contains fewer conserved motifs and a comparatively longer gene structure, which is consistent with its leaf-preferential expression and relatively strong transcriptional responses under abiotic stresses. This overall pattern is also consistent with recent genome-wide studies in soybean and other crops, which likewise reported conserved *MGT*/*MRS*2 family architecture together with species-specific functional diversification (Akter et al., 2025; Sarraf et al., 2026).

Chromosomal localization revealed an uneven distribution of *SiMGT* genes, with clustering on Chromosome 9, a pattern commonly observed in gene families and associated with functional redundancy or coordinated regulation. Collinearity analysis identified only one segmental duplication pair (*SiMGT*8/*SiMGT*9), indicating that *SiMGT* family expansion is driven by limited segmental duplication rather than whole-genome duplication, which differs from the expansion mechanisms reported in peach (Zhou et al., 2024) and cassava (Tan et al., 2024). These results suggest that the *SiMGT* family remained relatively compact during evolution while retaining the core structural features of plant *MGT* genes. Interspecific collinearity with maize and rice confirms the close evolutionary relationships among graminaceous crops, providing cross-species functional references for *SiMGT* genes. For instance, *SiMGT2* clusters with *OsMRS2-3*, a homolog reported to be involved in salt stress responses (Saito et al., 2013), and also shows strong salt-induced expression in the present study, suggesting potential functional conservation between these homologs. All *SiMGT* proteins contained the conserved Mrs2_Mfm1p-like domain, supporting the structural conservation of the *SiMGT* family, which is consistent with previous reports on MGT-related transporter domains (Sponder et al., 2013; Chen et al., 2017). Motif and gene structure analysis revealed that closely related phylogenetic members share similar motif arrangements and gene structures, supporting functional conservation, while differences (e.g., *SiMGT*7’s reduced motif number and expanded gene structure) imply potential functional divergence among family members. This “conserved core + divergent subfamilies” pattern is consistent with the evolutionary strategy of gene families to adapt to diverse environmental conditions (Wu et al., 2022). Cis-acting element analysis identified light-responsive elements in all *SiMGT* promoters, suggesting that some *SiMGT* members may be associated with light-responsive or photosynthesis-related processes, which aligns with the leaf-specific expression of *SiMGT*7 and the chloroplast localization of several *SiMGT* members. Stress-responsive and hormone-responsive elements further support the involvement of *SiMGT*s in abiotic stress adaptation and hormonal regulation, consistent with their expression patterns under drought, cold, salt, and ABA treatments. In particular, ABA was included in this study because it is a central phytohormone in abiotic stress signaling, and previous work has suggested that ABA signaling also participates in Mg-related stress responses (Zhu, 2016; Guo et al., 2014). The widespread occurrence of ABRE elements in *SiMGT* promoters, together with the rapid induction of *SiMGT*1, *SiMGT*2, and *SiMGT*3 under ABA treatment, supports a potential association between *SiMGT* regulation and ABA-mediated stress signaling.

Tissue-specific expression patterns indicate that constitutively expressed *SiMGT* genes (*SiMGT*1, *SiMGT*3, *SiMGT*6, *SiMGT*7, *SiMGT*8) maintain basal Mg²^+^ homeostasis, while organ-specific genes (*SiMGT*7 in leaves, *SiMGT*6 in panicles) fulfill tissue-specific physiological needs. Under abiotic stresses, *SiMGT* genes exhibit temporal and functional differentiation:*SiMGT7* and *SiMGT9* showed relatively strong responses to drought and cold stress, *SiMGT2* responded prominently to salt treatment, and *SiMGT1–3* were rapidly induced at the early stage of ABA treatment. These distinct transcriptional patterns suggest that different SiMGT members may contribute unequally to abiotic stress adaptation (Ma et al., 2016; Yang et al., 2020). For example, *SiMGT4* showed the highest induction under low temperature, indicating that it may participate in cold-responsive regulation; however, its direct effects on Mg²^+^ transport, chloroplast function, and photosynthesis remain to be validated experimentally.

Superior haplotypes of *SiMGT*6 and *SiMGT*7 associated with yield traits and stress resistance provide practical molecular markers for foxtail millet breeding, bridging the gap between gene function and crop improvement. Notably, *SiMGT*6 Hap1 is associated with increased panicle yield, while *SiMGT*7 Hap1 enhances stress tolerance, suggesting potential value for developing high-yield, stress-resilient foxtail millet varieties. However, this study has limitations: functional validation of *SiMGT* genes (e.g., via gene editing or overexpression) and exploration of their interactions with other ion transporters (e.g., Ca²^+^ transporters) are lacking, and no direct quantification of Mg²^+^ or other divalent ions by AAS or ICP-MS was included in the current work. In addition, no Mg²^+^-, Ca²^+^-, or other divalent-cation-specific treatments, and no physiological or biochemical indicators such as chlorophyll content, were examined in the present study. Furthermore, while in silico analyses provided valuable insights into the subcellular localization of SiMGT proteins, the lack of *in vitro* or *in vivo* experimental validation (such as GFP-fusion transient expression assays) represents a limitation of the current study. Therefore, these localization patterns should be strictly regarded as predictions, and experimental verification will be a priority in our upcoming functional characterization studies. Therefore, the proposed roles of *SiMGT* members in Mg²^+^ homeostasis and photosynthesis-related processes should be regarded as candidate inferences rather than direct functional proof. Additionally, post-transcriptional regulation of *SiMGT* genes, such as phosphorylation and miRNA targeting, warrants further investigation to fully understand their regulatory networks.

## 5 Conclusion

In this study, nine *SiMGT* genes were identified in foxtail millet, which exhibit conserved evolutionary features with graminaceous crops and functional divergence. Tissue-specific expression patterns indicate that *SiMGT6* and *SiMGT7* are candidate genes potentially associated with panicle development and leaf-related functions, respectively. Under abiotic stresses, *SiMGT* genes showed distinct temporal expression responses, with *SiMGT*7 and *SiMGT*9 showing relatively strong responses to drought and cold stress, and *SiMGT*2 responding prominently to salt treatment. Superior haplotypes of *SiMGT*6 and *SiMGT*7 associated with favorable phenotypic traits provide valuable molecular markers for breeding stress-tolerant and high-yielding foxtail millet varieties. Taken together, these findings provide a useful framework for understanding the evolutionary characteristics and stress-responsive expression patterns of the *SiMGT* family in foxtail millet and provide references for the functional investigation and application of *MGT* genes in other cereal crops. Nevertheless, direct validation of ion homeostasis, photosynthesis-related physiological traits, and transporter function will be necessary in future studies to clarify the precise roles of individual *SiMGT* members.

## Data Availability

The genome sequence, protein sequences, and annotation files of foxtail millet used in this study are available from the MDSi multi-omics database (http://foxtail-millet.biocloud.net/home). The genotype and phenotype data of foxtail millet accessions are available from the Setaria DB database (http://111.203.21.71:8000/index.html). The raw qRT-PCR data generated in this study are available from the corresponding author upon reasonable request.
